# Contemporary management of locally advanced prostate cancer: a narrative review and expert perspective

**DOI:** 10.1007/s00345-026-06190-x

**Published:** 2026-01-23

**Authors:** Mona Kafka, Isabel Heidegger

**Affiliations:** Department of Urology, Medical University Innsbruck, Anichstreet 35, 6020 Innsbruck, Austria

**Keywords:** Locally advanced prostate cancer, Surgery, Radiation, Systemic therapy, Personalized treatment

## Abstract

**Background:**

Locally advanced prostate cancer (PCa) encompasses a heterogeneous group of patients, including those with clinical stage T3–T4 disease and/or regional lymph node involvement (cN1), without distant metastases. While curative-intent treatment remains the goal in many cases, optimal management strategies are still subject to debate due to limited high-level evidence and variability in clinical guidelines.

**Objective:**

This narrative review summarizes current treatment guidelines, appraises recent clinical evidence, and offers an expert perspective on contemporary management strategies for patients with locally advanced PCa.

**Methods:**

A narrative review of international guidelines and recent clinical trials was conducted, focusing on the role of external beam radiotherapy (EBRT), androgen deprivation therapy (ADT), radical prostatectomy (RP), and systemic treatment intensification using androgen receptor pathway inhibitors (ARPIs) and chemotherapy. In addition, we highlight the impact of novel imaging modalities such as PSMA-PET/CT.

**Results:**

The established standard of care remains EBRT combined with ADT, with the addition of abiraterone now recommended for selected high-risk cases. RP, although not yet supported by randomized trials, may provide oncological and functional benefits in carefully chosen patients and is increasingly being incorporated into multimodal treatment strategies. The role of intensified neoadjuvant or adjuvant systemic therapies, however, remains under investigation.

**Conclusion:**

Current management of locally advanced PCa requires individualized decision-making based on modern imaging modalities, patient characteristics, tumor biology, and available resources. While EBRT based multimodal concepts are mainly the preferred treatment, RP based multimodal concepts play an important role in selected cases. Emerging systemic therapies and advanced imaging may further refine treatment strategies in the near future.

## Introduction

Prostate cancer (PCa) is the second most common malignancy in men worldwide, the most frequently diagnosed cancer in Europe, and the third leading cause of cancer-related deaths among males. In Western countries, educational campaigns and various early PCa detection programs, primary based on regular prostate-specific antigen (PSA) measurements, resulted in earlier PCa detection, followed by a higher likelihood of curative treatment in the majority of patients. According to data from the German cancer registry, at the time of primary diagnosis, 10–15% of patients already present with metastatic disease, while 70–75% have localized disease, and approximately 15–20% are diagnosed with locally advanced PCa [1].

Locally advanced PCa is defined as clinical stage T3-T4, indicating palpable tumor extension beyond the prostate capsule, regardless of PSA level or ISUP grade [2, 3]. In addition, patients with regional lymph node involvement (cN1) but no distant metastases are categorized as having locally advanced disease, irrespective of their clinical T-stage [2, 3]. This definition therefore encompasses a heterogeneous group of patients with varying PSA levels, ISUP grades, and nodal involvement, of notice based on conventional imaging modalities such as computer tomography (CT)/ magnetic resonance imaging (MRI) and bone scans.

In contrast to treatment of localized PCa, there is a less high-level evidence regarding effectiveness of local therapies in patients with locally advanced disease, particularly in those with cN1 status. Currently, the only available randomized phase III trials have evaluated external beam radiotherapy (EBRT) in combination with androgen deprivation therapy (ADT) while data on radical prostatectomy (RP) are primarily based on studies assessing surgery as part of a multimodal treatment strategy (MMT) [2]. However, despite the absence of robust data from randomized trials supporting surgery, RP offers specific advantages, including precise pathological staging through whole-gland histological analysis, which can improve prognostic accuracy and risk stratification for recurrence. It may also prevent local complications associated with extensive tumor growth like macrohematuria, pain, subvesical obstruction or hydronephrosis, and mitigate long-term adverse effects (AEs) of radiation. Moreover, studies have reported tumor persistence in up to 25% of patients following EBRT, which may contribute to an increased risk of recurrence and metastatic progression [4].

Given these uncertainties, we conducted a comprehensive narrative review summarizing current strategies and the optimal modern management of patients with locally advanced PCa.

## Evidence acquisition

We conducted a narrative literature review until July 2025 using PubMed and Google Scholar, focusing on studies in English that addressed the management of locally advanced PCa, including both node-negative (cN0) and node-positive (cN1) non-metastatic cases. In addition, we reviewed abstracts and presentations from major international urological and oncological conferences held over the last five years, including the American Society of Clinical Oncology (ASCO), ASCO Genitourinary Cancers Symposium (ASCO GU), the European Association of Urology (EAU), the European Society for Medical Oncology (ESMO), the American Urological Association (AUA), NCCN, S3—until the ASCO meeting 2025 meeting in June 2025. Included were clinical trials, guideline updates, meta-analyses, retrospective cohort studies, and expert opinions focusing on treatment modalities such as EBRT, ADT, RP (with or without extended lymph node dissection), and systemic treatment intensification strategies using androgen receptor pathway inhibitors (ARPIs) or chemotherapy. We also considered literature addressing the role of PSMA-PET/CT imaging in staging and response assessment (see Table 1).

**Table 1:** Overview of the patient group defines as locally advanced prostate cancer

Clinical stage	T3–T4
Tumor extension	Palpable tumor growth beyond the prostate capsule
PSA level	Not decisive for classification
ISUP grade	All ISUP grades; Not decisive for classification
Lymph node status (cN)	cN1: Regional lymph node metastases
Distant metastases (M)	No distant metastasis (M0)
Imaging modalities	Conventional staging with CT/MRI and bone scintigraphy
Patient collective	Heterogenic patient collective, including patients with different PSA levels, ISUP grades and possible regional lymph node involvement

### Guideline recommendations

According to current guidelines, EBRT combined with long-term ADT is generally considered the standard of care (SOC) for patients with locally advanced PCa [2, 3]. This approach is particularly emphasized in the subgroup of patients with cN1 disease, for whom EBRT combined with long-term ADT is the preferred treatment option. However, specific recommendations regarding the optimal duration of ADT in this setting remain unclear. Since the publication of the STAMPEDE trial (Arm G) in 2021, treatment intensification with the addition of abiraterone acetat/prednisolone for two years alongside long-term ADT (3 years) has been recommended for patients with cN1 disease and those with specific high-risk features (see Table 2) [5].

However, RP with extended pelvic lymph node dissection (ePLND) is also included in current guidelines, but is generally recommended only for selected patients with good performance status (PS), a long-life expectancy, and typically as part of a MMT approach. This may include adjuvant (ART) or salvage radiotherapy (SRT) and ADT, depending on the final pathological findings (see Table 2).

**Table 2:** Overview of the current guideline recommendations on the treatment of local advanced PCa [2, 3, 6–9]

Guideline	Recommendation
EAU (2024)	- EBRT + long-term ADT (2–3 year) is SOC - EBRT + long-term ADT + abiraterone for 2 year for cN0M0 patients with ≥ two high-risk factors (cT3-4, Gleason ≥ 8 or PSA ≥ 40 ng/mL) - EBRT to the prostate plus pelvis + long-term ADT + 2 years of abiraterone to cN1M0 patients - RP + ePLND in well-selected cases, combined with adjuvant/salvage RT and ADT depending on pathology
S3 (2025)	- EBRT + long-term ADT (2–3 years) is standard for cT3–T4 and cN1 M0 patients - RP + ePLND may be considered in well-selected cases, followed by adjuvant/salvage RT - In cN1 patients additional 2 year of abiraterone should be recommended
NCCN (2024)	- EBRT + long-term ADT for high-risk, clinically localized/locally advanced disease - ADT + abiraterone is recommended in very high-risk non-metastatic prostate cancer as the backbone systemic therapy with EBRT - RP ± PLND may be considered in high-risk or very high-risk patients (e.g., cT3a/b, N0, M0) who are: medically fit, suitable for multimodal therapy, treated in experienced canters
AUA/ASTRO/SUO (2022–2023)	- For high-risk localized cases: EBRT + long-term ADT (18–36 months) is strongly recommended - RP for selected patients with locally advanced PCa (e.g., cT3a-T3b, N0,M0) only as part of a multimodal approach

These recommendations again allow for a wide range of interpretation and practice patterns as duration on ADT isn’t clearly defined and there is no specific recommendation on the optimal peri- and post-operative strategy. Moreover, they do not yet fully incorporate the potential benefits of modern diagnostic technologies such as Prostate-specific membrane antigen (PSMA)-positron emissions tomography (PET)/CT. With this extremely sensitive technology, we now have a tool for improved patient selection — to identify patients with purely locally advanced PCa, detect minimal lymph node involvement, and enable reliable post-operative follow-up in patients with undetectable or low PSA levels, thus allowing for early salvage treatment of metastases.

### Role of EBRT

As previously mentioned, evidence from randomized controlled trials (RCTs) supports the role of EBRT with long-term ADT as the treatment of choice in patients with locally advanced PCa. However, it is important to note that these recommendations are primarily based on older trials, such as SPCG-7, EORTC 22,863, and RTOG 85 − 31, in which ADT monotherapy or EBRT alone served as comparators [10–12]. In these studies, long-term ADT, typically administered for three years, demonstrated superior outcomes compared to short-term ADT over six months [13]. It is noteworthy that these trials were conducted exclusively in patients without lymph node involvement (cN0).

In the RTOG85-31 trial, which investigated the use of additional ADT to planed EBTR, patients were classified according to their status and extent of lymph node involvement, and radiation was not only applied to the prostate but also to the lymphatic area (in correlation to the extent of lymph node involvement) in patients with positive lymph nodes [12]. Further randomized evidence for patients with cN1 disease, is lacking. Current support for EBRT in this subgroup stems largely from retrospective analyses, which nevertheless indicate a benefit for combining local therapy with long-term ADT. This combined approach had been the prevailing SOC until the publication of the STAMPEDE analysis in 2021 [5]. This trial introduced a treatment intensification strategy involving the ARPI, abiraterone, for two years in combination with three years of ADT and local prostate-directed EBRT in patients with cN1 disease or in cN0 patients presenting with at least two of the following three high-risk factors: clinical stage T3/T4, Gleason score 8–10, or PSA ≥ 40 ng/ml [14–16]. The trial demonstrated a distinct clinical benefit, with a 6-year metastasis-free survival (MFS) of 82% in the combination therapy group compared to 69% in the control group, prompting a shift in the SOC for this patient population. The addition of enzalutamide to this intensified regimen of the STAMPEDE trial did not further improve oncological outcomes. With respect to toxicity, grade ≥ 3 AEs occurred in 37% of patients in the combination arm versus 29% in the control group within 24 months, with hypertension and elevated alanine transaminase being the most frequently reported AEs [5].

Given that ARPIs have transformed the management of metastatic castration-sensitive PCa (mCSPC), their use is now being explored in earlier stages of disease. In this context, results from the randomized, double-blind, placebo-controlled phase III ATLAS trial—evaluating apalutamide in combination with ADT and EBRT in patients with high-risk localized or locally advanced PCa—are highly anticipated [17]. A similar trial, named ENZARAD, investigating enzalutamide in combination with ADT and EBRT is also ongoing [18]. Both trials are active, recruitment is already finished, and planned end of the trials is ENZARAD is March 2026 and December 2028 of the ATLAS trial. However, to date, no definitive recommendations can be made, and the use of ARPIs in this setting should still be restricted to clinical trials [17, 18].

Additional approaches for systemic treatment intensification have included the addition of chemotherapeutic agents such as docetaxel to ADT. Trials such as GETUG-12 and another arm of the STAMPEDE study have evaluated this combination and the NCCN guidelines currently suggest to considering six cycles of docetaxel in addition to ADT and EBRT for patients with very high-risk PCa [6, 19, 20]. Nonetheless, an update of the phase III RTOG 0521 trial—presented at the 2020 Genitourinary Cancers Symposium—showed only a modest improvement in overall survival (OS) (10-year OS: 69% vs. 64%) with the addition of docetaxel [21]. Therefore, at present, there is no strong evidence supporting the routine use of chemotherapy in this setting.

### Role of surgery- based multimodal approaches

Despite the lack of data from prospective randomized trials, current guidelines still recommend RP as part of a MMT strategy in patients with locally advanced, node-negative (cN0) PCa (see Table 1) [2]. For those undergoing surgery, ePLND is advised. In this patient cohort, long-term outcomes are encouraging, with reported cancer-specific survival (CSS) rates exceeding 60% at 15 years and OS rates surpassing 75% at 10 years [22–28]. However, there is no established consensus on the optimal perioperative or postoperative treatment strategy in this population. Clinically relevant questions remain regarding the role of neoadjuvant or adjuvant systemic therapy, the necessity and timing of postoperative radiotherapy, and how these elements should be standardized. In contrast to many other solid tumors, neoadjuvant approaches have not yet gained traction in PCa. It is noteworthy that the EAU Guidelines specifically recommend against neoadjuvant ADT in the pre-surgical setting [2].

In recent years, several phase II trials have investigated the effects of neoadjuvant ADT, primarily enrolling patients with high-risk PCa. However, to date, these studies have not demonstrated any explicit clinical benefit [29]. The only guideline-based recommendation for adjuvant ADT after RP in patients with undetectable PSA levels applies to those patients with pathologically confirmed lymph node involvement (pN1) and is based on 2 prospective RCTs. However it is important to note that that mostly patients with high-volume nodal disease and adverse tumor characteristics were included into these trials which should be kept in mind for patients selection [30, 31].

With regard to the role of RT, there remains ongoing debate surrounding the use of adjuvant radiotherapy (ART) versus early salvage radiotherapy (SRT) following RP. This question has been primarily addressed in three prospective randomized controlled trials—RADICALS-RT, RAVES, and GETUG-AFU 17 [32–34]. So far, primary endpoint data have been fully published only for the RADICALS-RT trial, which found no statistically significant difference in biochemical progression-free survival (bPFS) or OS after a median follow-up of 7.8 years [32]. Similarly, the ARTISTIC meta-analysis, which combined available, preliminary data from all three trials (even if follow-up has not been completed), showed no significant difference in oncological outcomes between ART and SRT at median follow-up times of 4.9 and 6.2 years, respectively [35]. However, it is important to emphasize that these trials underrepresented patients with high-risk pathological features—such as ISUP grade 4–5, pathological stage pT3, and positive surgical margins (R1). Consequently, the generalizability of these findings to such high-risk subgroups is limited, and recommendations in favour of SRT over ART should be interpreted with caution until more specific data are available.

Supporting this caution, a large retrospective analysis of over 26,000 patients—including 2,424 with high-risk features—found a lower acute mortality risk in those treated with ART [36]. Based on this and other evidence, ART is currently recommended in patients with ISUP grade 4–5 and pT3 disease, with or without R1 status [36]. In patients with pN1 disease, certain subgroups appear to benefit particularly from adjuvant RT: those with 3–4 positive lymph nodes, as well as those with ≤ 2 positive nodes in the presence of high-risk features such as ISUP grade 2–5, pT3–4 stage, or R1 [37].

A systematic review further supports the use of pelvic radiotherapy in patients with locally advanced PCa and multiple positive lymph nodes (in ePLND), suggesting that RT targeting the pelvic lymph nodes and prostate bed, combined with long-term ADT, can improve survival. Alternatively, for patients with ≤ 2 positive lymph nodes following ePLND, observation with deferred treatment maybe a viable strategy, as approximately 37% remained metastasis-free at 60 months of follow-up [38]. According to their analysis, higher pT-stage (pT2/3a vs. pT3b/4) and higher Gleason Score (GS) (≤ 7 vs. ≥8) were the two factor which strongest negatively influenced oncological outcome [38].

With regard to other modalities of systemic treatment intensification, several studies have evaluated the role of docetaxel in the neoadjuvant setting. One of the key trials in this context is the phase III PUNCH trial, which compared neoadjuvant docetaxel (6 cycles 75 mg/Kg/KOF) plus ADT followed by RP with ePLND versus immediate RP with ePLND [39]. While an improvement in MFS was observed, no benefit was seen in 3-year biochemical-free survival (bPFS) [39]. Two more recent, though smaller, trials also demonstrated promising findings: one reported improved pathological response rates, while the other showed prolonged 3-year bPFS with neoadjuvant docetaxel [40, 41].

In addition, the role of ARPIs in the neoadjuvant setting has been the subject of increasing research interest. Studies combining apalutamide with ADT have reported pathological complete response (pCR) rates of up to 14%, and minimal residual disease (MRD)—defined as ≤ 5 mm of residual tumor—was observed in 38% of patients [42, 43]. Furthermore, a strong correlation was found between favourable pathological responses and reduced biochemical recurrence (BCR) following intensified neoadjuvant therapy with apalutamide and/or enzalutamide, with a median follow-up of 3.6 years [44].

By contrast, ARPI monotherapy without concurrent ADT demonstrated inferior outcomes. For example, in the NEAR trial, 12 weeks of neoadjuvant apalutamide prior to RP resulted in limited response compared to combination regimens [45]. Similarly, the so-called “super-intensification” approach—combining ADT with two ARPIs in the neoadjuvant setting—showed a trend toward improved pathological outcomes, although statistical significance was not reached [46, 47]. Despite these early signals, long-term survival data for neoadjuvant intensified systemic therapy remain limited and inconclusive, and extended follow-up is needed before definitive conclusions can be drawn.

Regarding adjuvant systemic treatment intensification beyond ADT, several trials have investigated agents such as docetaxel, mitoxantrone, and ARPIs. However, results so far have been largely disappointing [29]. One notable exception, is a recently published study, involving 108 men with high-risk localized disease. In this trial, 12 weeks of adjuvant ADT combined with apalutamide following RP resulted in a 2-year bPFS of 100% [48].

## Discussion

The treatment landscape for PCa has undergone substantial changes in recent years. Beyond the well-documented paradigm shift in the systemic management of metastatic disease, therapeutic strategies for locally advanced and oligometastatic PCa are also evolving. Historically, RP was often withheld in cases of lymph node involvement or high PSA levels. Today, however, these patient subgroup may benefit from curative-intended treatment.

The broad definition of locally advanced PCa, encompassing a wide spectrum of tumor stages, risk profiles, and imaging findings, allows for diverse therapeutic approaches. Consequently, treatment strategies vary significantly between specialized medical centres. Decision-making depends heavily on individual patient characteristics such as ISUP grade, PSA level, pelvic MRI findings, nodal status, age, life expectancy, and as well as the treating physician’s specialty (urology, radiation oncology, or medical oncology).

As discussed above, high-level evidence currently supports EBRT combined with long-term ADT as the SOC for patients with locally advanced disease. Nevertheless, in our opinion, a subset of patients—particularly younger individuals with high-risk features—may benefit significantly from RP. RP offers the potential to avoid or at least delay long-term ADT and its associated side effects as osteoporosis, weight gain, and cardiotoxicity as well as restrictions in sexuality which lead to a reduced quality of life (QoL).

Furthermore, the implementation of PSMA-PET/CT has added considerable value to both, primary staging and post-treatment monitoring. With its superior sensitivity for detecting metastatic lesions, PSMA-PET/CT enables more accurate identification of patients with early or low-volume metastatic spread (e.g., cN1 disease). It is also highly effective in detecting recurrence at low PSA levels during postoperative follow-up, with reported detection rates of 38% at PSA < 0.5 ng/ml and 54% at PSA < 1 ng/ml. This allows for timely implementation of local salvage therapies—such as early SRT or metastasis-directed surgery—potentially deferring the need for systemic treatment [49]. Additionally, nowadays we also have the option of enzalutamide with or without ADT in non-metastatic patients with BCR and a high risk for metastatic progression since the presentation of the EMBARK trial [50]. Conversely, PSMA-PET/CT also helps to more accurately identify patients with true metastatic disease who are best managed with upfront systemic therapy. Notably, the PEACE-1 trial demonstrated that the addition of local treatment (EBRT) to systemic therapy significantly improved outcomes in patients with de novo low-volume metastatic PCa, based on conventional imagine [51]. Although this trial focused on EBRT, it is reasonable to hypothesize that other forms of local therapy, as RP, might confer similar benefits in appropriately selected patients.

In support of local treatment, several population-based analyses have demonstrated improved OS and CSS in patients with cN1 disease who received local therapy (surgery or EBRT) compared to those managed with ADT alone [52, 53]. While ADT monotherapy is no longer an appropriate comparator by contemporary standards, these data highlight the importance of treating the primary tumor, even in node-positive patients.

The question of the value of RP versus primary EBRT combined with ADT in this setting remains unanswered so far. The ongoing phase III SPCG-15 trial is the first prospective randomized trial to directly compare RP with ePLND (plus adjuvant or salvage RT ± ADT) to EBRT combined with long-term ADT [54]. Recruitment is ongoing since 2014, and the primary results are expected in 2027. However, it must be acknowledged that the control arm in SPCG-15 may already be outdated, given that intensified treatment with two years of abiraterone plus three years of ADT is now recommended for patients with at least two of three high-risk features (cT3/4, Gleason score ≥ 8, or PSA ≥ 40 ng/ml), based on the STAMPEDE Arm H trial and also advantages through the implementation of PSMA-PET/CT are not included [55]. Complementary to this topic, a recent presentation at ASCO GU 2025 by Soumyajit et al. offered an emulated randomized comparison using data from two independent phase III trials: NRG/RTOG 0521 (EBRT + long-term ADT ± 6 cycles of docetaxel) and CALGB 90,203 (RP ± neoadjuvant docetaxel and ADT) [39, 56, 57]. The analysis included 1,290 high-risk patients. Results showed that EBRT-based treatment was associated with a significantly lower incidence of distant metastases after approximately 8 years of follow-up (16% vs. 23%, hazard ratio [HR]: 0.56). A similar benefit was observed when comparing personalized EBRT + ADT to RP followed by postoperative individualized therapy (16% vs. 26%, HR: 0.59). Notably, only when neoadjuvant docetaxel + ADT was administered prior to RP did the difference in distant metastasis rates narrow substantially (18% vs. 22%, HR: 0.84). Although these findings support the use of EBRT in patients with high-risk localized PCa, it is important to emphasize that the comparison was emulated and not based on a direct randomized trial. Limitations include a relatively short follow-up period, a modest number of clinical events, and the lack of integration of advanced imaging modalities such as PSMA-PET/CT, which could significantly influence postoperative management and outcomes in our opinion. On the other hand, a recently published SEER database analysis of 9215 newly diagnosed metastatic PCa patients, including 371 patients undergoing RP as local therapy, found a significant higher CSS rate (3 year CSS 76.5% no local treatment vs. 90.2% RP) in the group of patients undergoing RP compared to no local treatment, except patients M1c or PSA ≥ 60ng/ml [58]. Although this was a retrospective study and was focused on metastatic patients, it supports the hypothesis that treatment of the primary tumor might have a beneficial effect on PFS/OS even in with patients with low-volume metastatic disease.

**Fig. 1:**
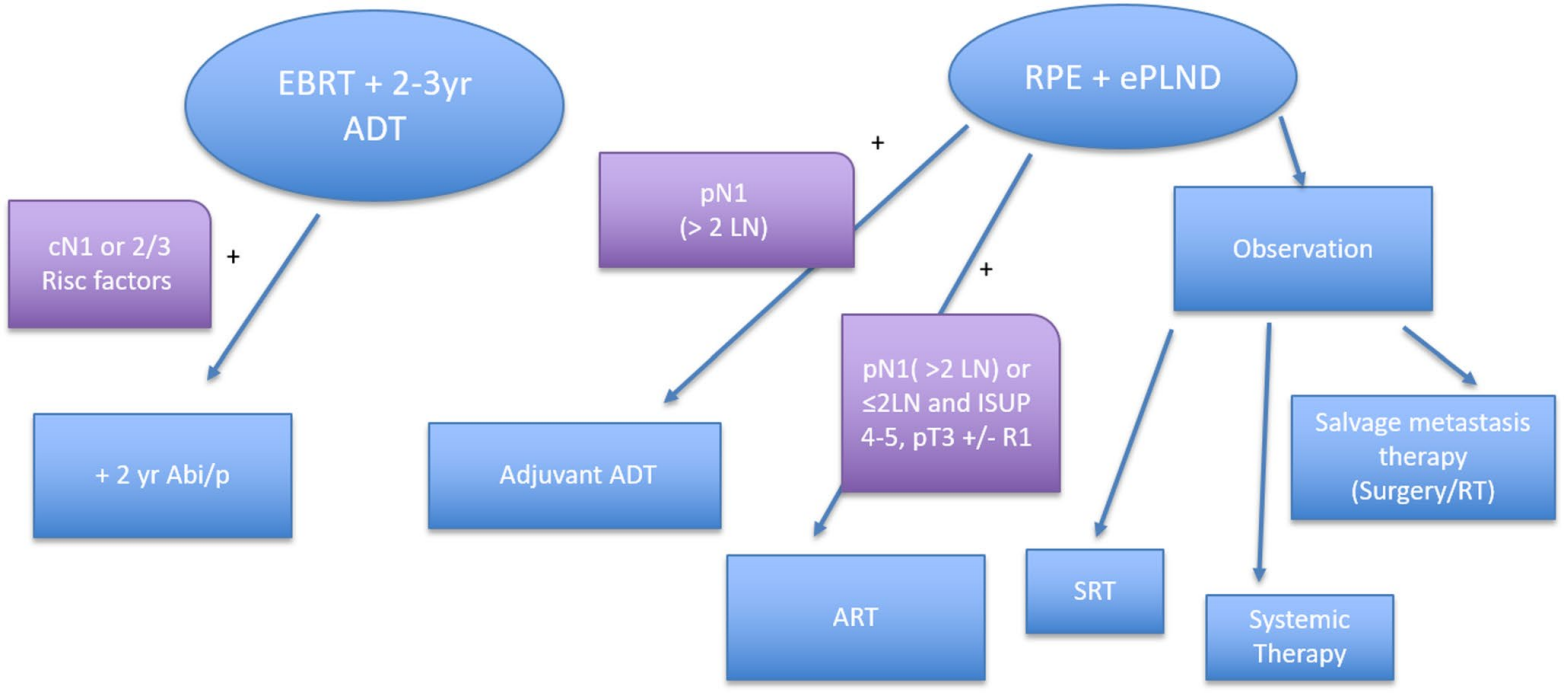
Flow-chart depicting potential treatment strategies for local advanced PCa. *LN* lymph node, *SRT* salvage RT, *ART* adjuvant RT, *yr* years

## Conclusion

The management of locally advanced PCa continues to vary widely in clinical practice, largely due to the absence of uniformly clear guideline recommendations and the availability of diverse therapeutic strategies. Generally, high-level evidence for randomized clinical trials supports to use of EBRT in combination with long-term ADT, with or without the addition of abiraterone. However, given the heterogeneity encompassed by the definition of locally advanced PCa, we believe that RP still has an important role in carefully selected patients. In particular, the integration of highly sensitive imaging technologies such as PSMA-PET/CT allows better patient selection and precise postoperative monitoring. This may enable some patients to avoid or delay the initiation of systemic therapy—along with its well-known side effects—and thereby preserve QoL. While a good portion of patients will ultimately experience disease recurrence and require further treatment, a “watchful waiting” or deferred therapy approach after RP may be reasonable in well-informed, highly compliant individuals who prioritize a personalized treatment strategy. In selected cases, postponing the initiation of ADT could also potentially delay the onset of castration-resistant disease. Additionally, it should be acknowledged that also decision for EBRT is always a form of MMT as it accompanies long-term ADT and even abiraterone. Therefore, both treatment strategies, either RP or EBRT as form of local treatment, classify as MMT (Summarized in Fig. 1). In our opinion, treatment decision for this specific subpopulation of PCa should be made by a multidisciplinary tumor board in the sense of personalized medicine.

With regard to systemic treatment intensification in the neoadjuvant or adjuvant setting using chemotherapy or ARPIs, current evidence remains preliminary. Consequently, such strategies should only be pursued in clinical trial settings until reliable prospective data are obtained.

At least, we have to state some limitation of these work: first of all, this is not a systematic review but a narrative review of the literature, so relevant studies could be missed. Furthermore, we represent urologists which are treating patients in clinical practice, which might lead to a bias towards surgical approaches, even if unintended.

## Data Availability

No datasets were generated or analysed during the current study.
